# Noble-Gas Chemistry More than Half a Century after the First Report of the Noble-Gas Compound

**DOI:** 10.3390/molecules25133014

**Published:** 2020-07-01

**Authors:** Zoran Mazej

**Affiliations:** Department of Inorganic Chemistry and Technology, Jožef Stefan Institute, Jamova cesta 39, SI–1000 Ljubljana, Slovenia; zoran.mazej@ijs.si; Tel.: +386-1-477-3301

**Keywords:** helium, neon, argon, krypton, xenon, noble gases, fluorides, oxides

## Abstract

Recent development in the synthesis and characterization of noble-gas compounds is reviewed, i.e., noble-gas chemistry reported in the last five years with emphasis on the publications issued after 2017. XeF_2_ is commercially available and has a wider practical application both in the laboratory use and in the industry. As a ligand it can coordinate to metal centers resulting in [M(XeF_2_)_x_]^n+^ salts. With strong Lewis acids, XeF_2_ acts as a fluoride ion donor forming [XeF]^+^ or [Xe_2_F_3_]^+^ salts. Latest examples are [Xe_2_F_3_][RuF_6_]·XeF_2_, [Xe_2_F_3_][RuF_6_] and [Xe_2_F_3_][IrF_6_]. Adducts NgF_2_·CrOF_4_ and NgF_2_·2CrOF_4_ (Ng = Xe, Kr) were synthesized and structurally characterized at low temperatures. The geometry of XeF_6_ was studied in solid argon and neon matrices. Xenon hexafluoride is a well-known fluoride ion donor forming various [XeF_5_]^+^ and [Xe_2_F_11_]^+^ salts. A large number of crystal structures of previously known or new [XeF_5_]^+^ and [Xe_2_F_11_]^+^ salts were reported, i.e., [Xe_2_F_11_][SbF_6_], [XeF_5_][SbF_6_], [XeF_5_][Sb_2_F_11_], [XeF_5_][BF_4_], [XeF_5_][TiF_5_], [XeF_5_]_5_[Ti_10_F_45_], [XeF_5_][Ti_3_F_13_], [XeF_5_]_2_[MnF_6_], [XeF_5_][MnF_5_], [XeF_5_]_4_[Mn_8_F_36_], [Xe_2_F_11_]_2_[SnF_6_], [Xe_2_F_11_]_2_[PbF_6_], [XeF_5_]_4_[Sn_5_F_24_], [XeF_5_][Xe_2_F_11_][Cr^V^OF_5_]·2Cr^VI^OF_4_, [XeF_5_]_2_[Cr^IV^F_6_]·2Cr^VI^OF_4_, [Xe_2_F_11_]_2_[Cr^IV^F_6_], [XeF_5_]_2_[Cr^V^_2_O_2_F_8_], [XeF_5_]_2_[Cr^V^_2_O_2_F_8_]·2HF, [XeF_5_]_2_[Cr^V^_2_O_2_F_8_]·2XeOF_4_, A[XeF_5_][SbF_6_]_2_ (A = Rb, Cs), Cs[XeF_5_][Bi_x_Sb_1-x_F_6_]_2_ (x = ~0.37–0.39), NO_2_XeF_5_(SbF_6_)_2_, XeF_5_M(SbF_6_)_3_ (M = Ni, Mg, Zn, Co, Cu, Mn and Pd) and (XeF_5_)_3_[Hg(HF)]_2_(SbF_6_)_7_. Despite its extreme sensitivity, many new XeO_3_ adducts were synthesized, i.e., the 15-crown adduct of XeO_3_, adducts of XeO_3_ with triphenylphosphine oxide, dimethylsulfoxide and pyridine-N-oxide, and adducts between XeO_3_ and N-bases (pyridine and 4-dimethylaminopyridine). [Hg(KrF_2_)_8_][AsF_6_]_2_·2HF is a new example of a compound in which KrF_2_ serves as a ligand. Numerous new charged species of noble gases were reported (ArCH_2_^+^, ArOH^+^, [ArB_3_O_4_]^+^, [ArB_3_O_5_]^+^, [ArB_4_O_6_]^+^, [ArB_5_O_7_]^+^, [B_12_(CN)_11_Ne]^−^). Molecular ion HeH^+^ was finally detected in interstellar space. The discoveries of Na_2_He and ArNi at high pressure were reported. Bonding motifs in noble-gas compounds are briefly commented on in the last paragraph of this review.

## 1. Introduction

After almost 20 years, when the Christe’s paper “A renaissance in noble gas chemistry” appeared [1], we can say that the renaissance still lasts, although to a lesser extent than in the past. Times are changing, and with that, the fields of research in science. However, new topics are also present in noble-gas (Ng) chemistry. Beside classical Ng chemistry, which includes the syntheses of larger quantities of noble gas compounds (bulk-phase compounds), nowadays there are many other kinds of studies connected with the identification and characterization of molecules of Ng compounds. They include the preparations of Ng compounds in cold matrices, syntheses in liquid and supercritical noble gases, formations of Ng compounds under high pressures, and syntheses of neutral and gas-ion compounds in the gas phase [2]. Lastly, we should not forget to mention a large number of theoretical papers predicting new Ng compounds, which still need to be experimentally confirmed.

The last extensive review of Ng compounds was published in 2018 [2]. It gives a short overview of bulk-phase compounds, molecules of Ng compounds formed in cold matrices, molecules formed in liquid and supercritical noble gases, and the formation of Ng compounds under high pressures. Chapters that are more extensive are related to theoretical chemistry and to gas-phase chemistry of the noble gases. Before that reviews were given by Haner and Schrobilgen in 2015 (xenon(IV) compounds) [3], Nabiev et al. in 2014 (structures of Ng compounds) [4], Brock et al. in 2013 (general about Ng compounds) [5], and Hope in 2013 (coordination chemistry of Ng compounds) [6]. An extensive list of older references of Ng chemistry reviews can be found in the list of references given in the works of Saha et al. from 2019 [7], Grochala from 2018 [8], and Grandinetti from 2018 [2].

The present review is related to the synthetic Ng chemistry of some noble-gas compounds reported in the last five years, with emphasis on the examples reported after 2017, and few studies published before that, which were not mentioned in a 2018 review [2]. Since the present contribution is related to experimental synthesis, pure theoretical papers that predict new Ng compounds are not included. Concerning their number, a separate contribution can be written.

## 2. Xenon

Chemistry of xenon represents the field with the largest number of synthesized Ng compounds. Xenon(II) fluoride is the only Ng compound that is commercially available and has a wider practical application both in the laboratory use and in the industry. Its main use is in its ability of fluorination of various organic compounds [9,10] and as an etching reagent for surfaces of various metals, oxides, nitrides, etc. [11,12,13,14,15,16]. It is also used for the preparation of fluorographenes [17,18,19]. Its applicability for low-temperature insertion of fluorine into oxides systems has been demonstrated with the purpose of modification of magnetic and electronic properties, in particular superconductivity [20]. The ^18^F labeled XeF_2_ is used in nuclear medicine. This includes imaging techniques such as positron emission tomography (PET) as an ^18^F source [21]. Xenon is shown to bind to other molecules not only under matrix isolation (Lewis acid-base complex of 1,2-azaborine and Xe) [22] but also bulk-phases with metal-xenon bonds have been prepared and their crystal structures determined [23]. In a recent work from surface science, the Xe-Ru interactions with a significant amount of charge transfer were demonstrated at room temperature and low pressures [24]. Using new strategies taking advantage of confinement effects, these new two-dimensional structures allow the study of noble gas metal interactions down to the atomic scale, using the very sensitive toolkit of surface science methodology. The general ideas for using confinement effects for favoring interactions of noble gases with other elements at mild conditions is described in a recent topical review [25].

### 2.1. Xenon(II)

Since the first reported case of a compound with a XeF_2_ ligand coordinated to a metal center, i.e., [Ag(XeF_2_)_2_](AsF_6_) [26], a large number of other examples have been synthesized [27]. A variety of compounds exist, where different numbers of XeF_2_ molecules are bound to various metal centers yielding [M(XeF_2_)_x_]^n+^ cationic part. The oxidation states of the metals are M(I), M(II), or M(III). The most important aspects that influence the formation of XeF_2_ coordination compounds are charge and the size of the cation, the type of the anion that compensates the positive charge of the cationic part, solubility of the salt, and the concentration of the XeF_2_ ligand [27].

**Figure 1:**
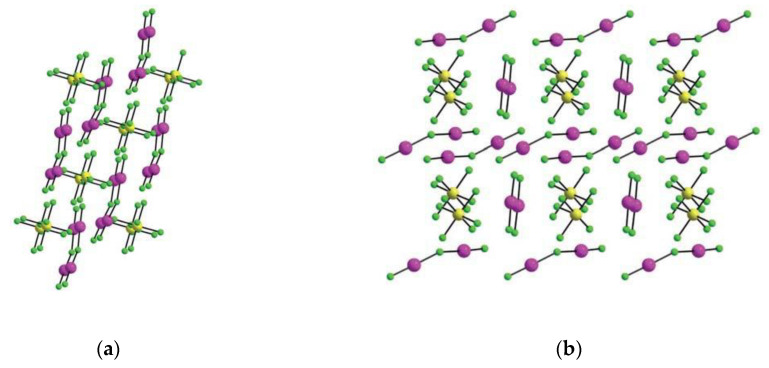
(**a**) Packing diagram of [Xe_2_F_3_][RuF_6_] along *b*-axis.; (**b**) Packing diagram of [Xe_2_F_3_][RuF_6_]·XeF_2_. (reproduced from Ref. [29]; published under the terms and conditions of the Creative Commons Attribution 4.0 International License CC BY 4.0.).

Beside acting as a neutral ligand, XeF_2_ acts as a fluoride ion donor forming [XeF]^+^ salts with strong Lewis acid pentafluorides (MF_5_; M = As, Sb, Bi, etc.) [28]. Although the ionic formulations, i.e., [XeF]^+^[MF_6_]^−^, implies complete F^−^ transfer from XeF_2_ to the strong Lewis axcid MF_5_, in reality the [XeF]^+^ cation and its anion exists as ion pairs in their salts that interact through Xe–F···M bridges [28]. An excess of XeF_2_ oftentimes results in the formation of [Xe_2_F_3_]^+^ salts. Oxidation of ruthenium and iridium metal with an excess of XeF_2_ in anhydrous HF as a solvent and further crystallization resulted in the crystal growth of [Xe_2_F_3_][RuF_6_]·XeF_2_, [Xe_2_F_3_][RuF_6_], and [Xe_2_F_3_][IrF_6_] (Figure 1) [29]. Crystal structure determination of [Xe_2_F_3_][MF_6_] (M = Ru, Ir) showed that their structures are isotypic.

**Figure 2:**
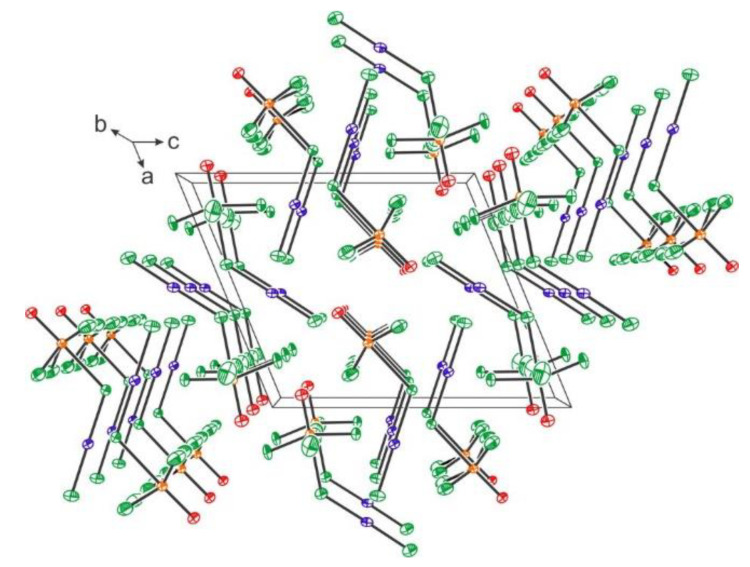
The crystal packing of XeF_2_·CrOF_4_ viewed along the *b*-axis of the unit cell. Copyright (2019) Wiley. Used with permission from Ref. [28].

The adducts XeF_2_·CrOF_4_ and XeF_2_·2CrOF_4_ were synthesized and structurally characterized at low temperatures (Figure 2) [28]. The fluoride ion affinity of CrOF_4_ is too low to enable fluoride ion transfer from XeF_2_ to form ion-paired salts of the [XeF]^+^ cations having either the [CrOF_5_]^−^ or [Cr_2_O_2_F_9_]^−^ anions [28].

### 2.2. Xenon(VI) Fluoride and its [XeF_5_]^+^ and [Xe_2_F_11_]^+^ Salts

XeF_6_ was prepared and isolated in solid argon and neon matrices [30]. The IR spectra of ^129^XeF_6_ and ^136^XeF_6_ isotopologues, recorded in the neon matrix, agree with theoretical ones for the C_3v_ conformer of the XeF_6_ molecule in the neon matrix. As an internal reference matrix-site and Xe-isotope splitting of corresponding Xe–F and Xe=O stretching modes of XeOF_4_ were analyzed in solid neon [30].

**Figure 3:**
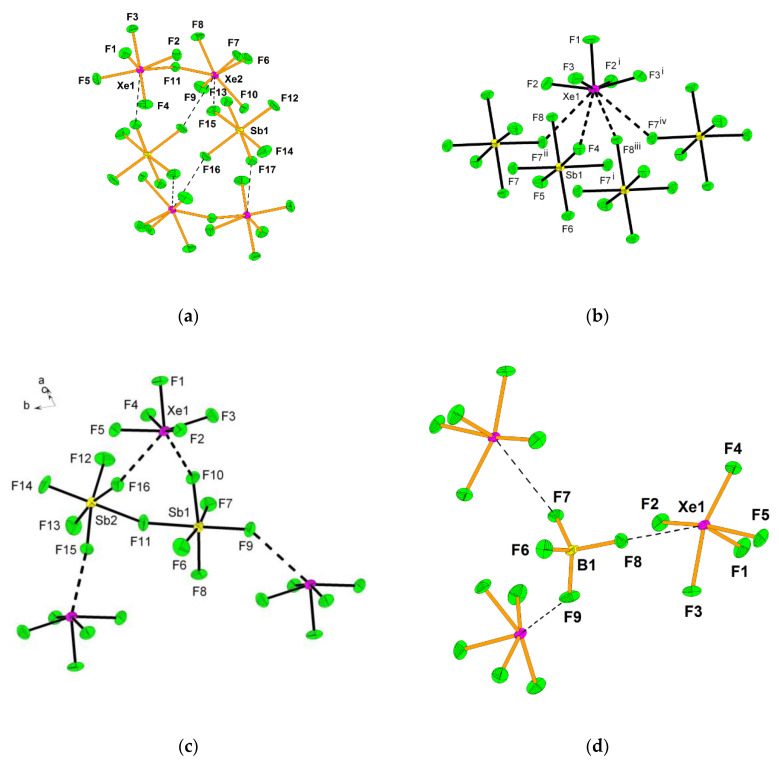
(**a**) Part of the crystal structure of [Xe_2_F_11_][SbF_6_]. Copyright (2017) Wiley. Used with permission from Ref. [32]; (**b**) Part of the crystal structure of the XeF_5_SbF_6_ showing the interactions between the [XeF_5_]^+^ cation and the [SbF_6_]^−^ anions. Copyright (2015) Elsevier. Used with permission from Ref. [33]; (**c**) Part of the crystal structure of XeF_5_Sb_2_F_11_ showing the interactions between the [Sb_2_F_11_]^−^ anion and the three [XeF_5_]^+^ cations. Copyright (2015) Elsevier. Used with permission from Ref. [33]; (**d**) Part of the crystal structure of [XeF_5_][BF_4_]. Copyright (2017) Wiley. Used with permission from Ref. [32].

Xenon hexafluoride is a well-known fluoride ion donor forming various [XeF_5_]^+^ and [Xe_2_F_11_]^+^ salts [31]. In recent years a large number of crystal structures of previously known or new [XeF_5_]^+^ and [Xe_2_F_11_]^+^ salts were reported. The crystal structure of [Xe_2_F_11_][SbF_6_] consists of [Xe_2_F_11_]^+^ cations and [SbF_6_]^−^ anions (Figure 3) [32]. The crystal structures of [XeF_5_][SbF_6_] [33], [XeF_5_][Sb_2_F_11_] [33], and [XeF_5_][BF_4_] [32] are all built from [XeF_5_]^+^ cations (Figure 3). Positive charge of the [XeF_5_]^+^ cations is compensated by [SbF_6_]^−^, [Sb_2_F_11_]^−^ or [BF_4_]^−^, respectively, anions (Figure 3).

**Figure 4:**
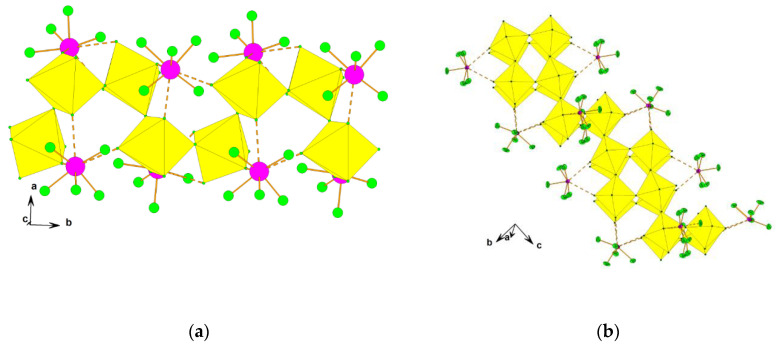
(**a**) Part of the ([TiF_5_]^−^)_∞_ chain in [XeF_5_][TiF_5_]; (**b**) Part of the infinite ([Ti_3_F_13_]^−^)_∞_ column in [XeF_5_][Ti_3_F_13_]. Ref. [35]; reproduced by permission of The Royal Society of Chemistry (RSC) on behalf of the Centre National de la Recherche Scientifique (CNRS) and the RSC.

Reactions between various amounts of XeF_2_ and MF_2_, MF_3_, or MF_4_ (M = Ti, Mn, Sn, Pb) with UV-photolyzed F_2_ in liquid anhydrous hydrogen fluoride (aHF) [34] resulted in the crystal growth of a large number of [XeF_5_]^+^ and [Xe_2_F_11_]^+^ salts upon crystallization from corresponding solutions. Investigation of XeF_2_/TiF_4_/UV-irradiated system resulted in the crystal structure determinations of [XeF_5_][TiF_5_] (XeF_6_·TiF_4_), [XeF_5_]_5_[Ti_10_F_45_] (XeF_6_·2TiF_4_), and [XeF_5_][Ti_3_F_13_] (XeF_6_·3TiF_4_) [35]. The main structural feature of [XeF_5_][TiF_5_] is an infinite chain of distorted TiF_6_ octahedra joined by cis vertices. Anionic part of [XeF_5_][Ti_3_F_13_] is built from tetrameric Ti_4_F_20_ and octameric Ti_8_F_36_ units sharing vertexes and alternatively linked into ([Ti_3_F_13_]^−^)_∞_ columns. In both cases the charge balance is maintained by [XeF_5_]^+^ cations which form secondary Xe···F contacts with fluorine atoms of anionic parts (Figure 4).

**Figure 5:**
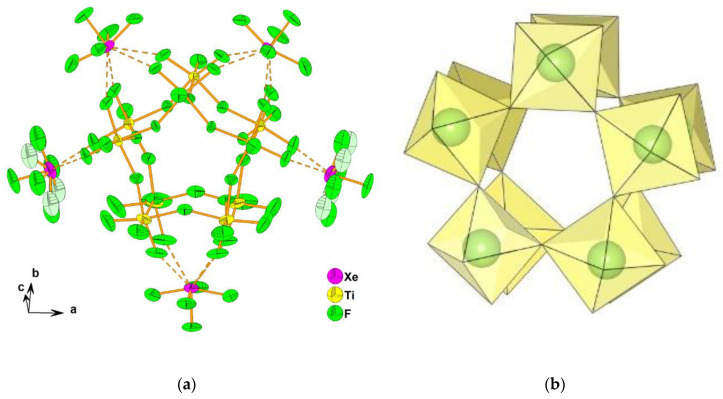
(**a**) [XeF_5_]^+^ cations and [Ti_10_F_45_]^5−^ anions, composed of ten TiF_6_ octahedra, in β-[XeF_5_]_5_[Ti_10_F_45_]. Ref. [35]; reproduced by permission of The Royal Society of Chemistry (RSC) on behalf of the Centre National de la Recherche Scientifique (CNRS) and the RSC; (**b**) Geometry of the [Ti_10_F_45_]^5−^ anion (Figure was made by Z.M. using crystallographic data from CIF-file of β-[XeF_5_]_5_[Ti_10_F_45_] [35]).

The crystal structure of [XeF_5_]_5_[Ti_10_F_45_] consists of [XeF_5_]^+^ cations and the largest known discrete [Ti_10_F_45_]^5−^ anion built from ten TiF_6_ octahedra, sharing vertices in the shape of a double-star (Figure 5). It crystallizes in two crystal modification at low (α-phase, 150 K) and ambient (β-phase, 296 K) temperatures.

**Figure 6:**
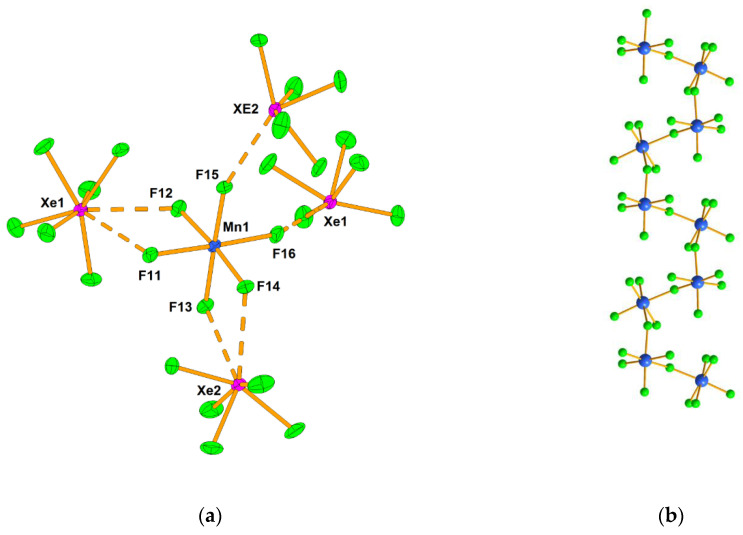
(**a**) The secondary contacts between the [MnF_6_]^2−^ anion and [XeF_5_]^+^cations in the crystal structure of [XeF_5_]_2_[MnF_6_]; (**b**) Part of the ([MnF_5_]^−^)_∞_ infinite chain in the crystal structure of [XeF_5_][MnF_5_]. Copyright (2017) Wiley. Used with permission from Ref. [36].

Photochemical synthesis between XeF_2_, MnF_3_, and UV-irradiated elemental F_2_ in aHF yielded [XeF_5_]_2_[MnF_6_] (2XeF_6_·MnF_4_), [XeF_5_][MnF_5_] (XeF_6_·MnF_4_) and [XeF_5_]_4_[Mn_8_F_36_] (XeF_6_·2MnF_4_) [36]. The crystal structure of [XeF_5_]_2_[MnF_6_] consists of [XeF_5_]^+^ cations and octahedral [MnF_6_]^2−^ anions (Figure 6).

The main structural feature of the anionic part in [XeF_5_][MnF_5_] is similar to that in [XeF_5_][TiF_5_] (Figure 4a), although the compounds are not isotypic (Figure 6b). A study of magnetic properties showed that [XeF_5_][MnF_5_] is paramagnetic in the 296–200 K range (*μ*_eff_ = 3.87 *μ*_B_, *θ* = −9.3 K). Below 100 K, there is a weak antiferomagnetic coupling between the Mn(IV) ions (*J* = −1.3 cm^−1^) [36].

**Figure 7:**
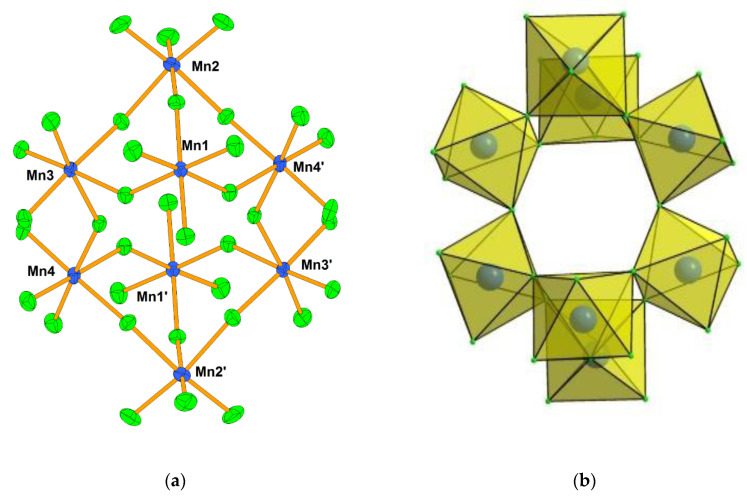
(**a**) Discrete octameric [Mn_8_F_36_]^4−^ anion in the crystal structure of [XeF_5_]_4_[Mn_8_F_36_]; (**b**) Geometry of the [Mn_8_F_36_]^4−^ anion built from eight MnF_6_ octahedra. Copyright (2017) Wiley. Used with permission from Ref. [36].

Crystal structure of [XeF_5_]_4_[Mn_8_F_36_] consists from [XeF_5_]^+^ cations and discrete [Mn_8_F_36_]^4−^ anions (Figure 7). The latter are built from eight MnF_6_ octahedra, each sharing three vertices, in the shape of a ring (Figure 7).

**Figure 8:**
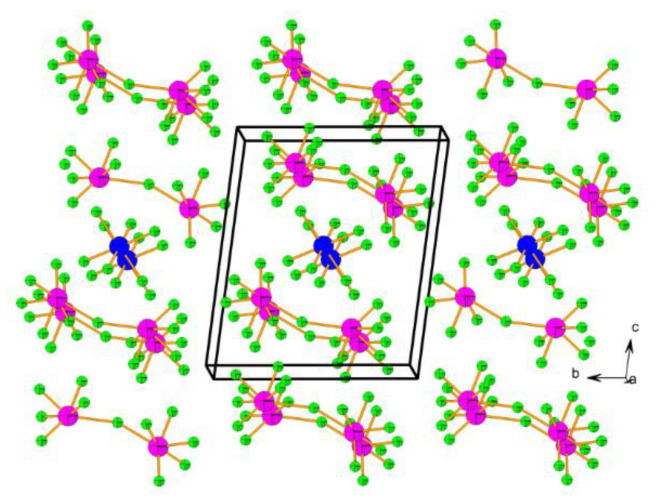
Packing of the [Xe_2_F_11_]^+^ cations and [MF_6_]^2−^ anions in the crystal structures of [Xe_2_F_11_]_2_[MF_6_] (M = Pb, Sn). Copyright (2019) Wiley. Used with permission from Ref. [37].

The attempts to grow single crystals in XeF_6_/MF_4_ (M = Sn, Pb) system were successful in three cases. Single crystals of [Xe_2_F_11_]_2_[SnF_6_], [Xe_2_F_11_]_2_[PbF_6_], and [XeF_5_]_4_[Sn_5_F_24_] were grown from aHF saturated solutions upon crystallizations [37]. The crystal structures of [Xe_2_F_11_]_2_[SnF_6_] and [Xe_2_F_11_]_2_[PbF_6_] are isotypic. They consist of [Xe_2_F_11_]^+^ cations and [MF_6_]^2−^ (M = Sn, Pb) anions (Figure 8).

**Figure 9:**
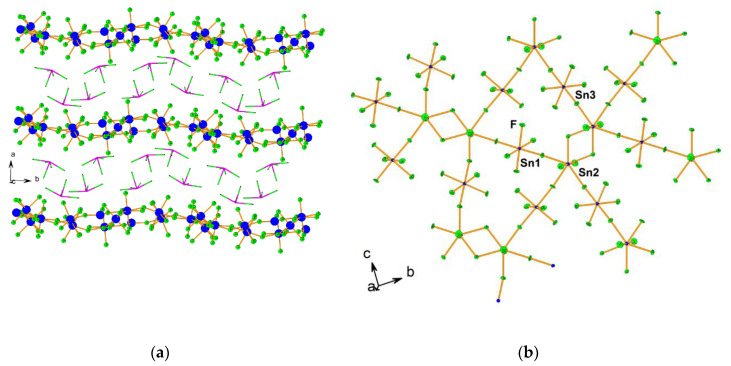
(**a**) Two-dimensional ([Sn_5_F_24_]^4−^)_∞_ grids with a wave-like conformation and the [XeF_5_]^+^ cations located between them in the crystal structure of [XeF_5_]_4_[Sn_5_F_24_]. For clarity, the [XeF_5_]^+^ cations are presented with sticks only; (**b**) The ([Sn_5_F_24_]^4−^)_∞_ layer in the crystal structure of [XeF_5_]_4_[Sn_5_F_24_] contains both six- and seven-coordinated Sn(IV) interconnected by bridging fluorine atoms (view perpendicular to the layer, along the *a*-axis). Copyright (2019) Wiley. Used with permission from Ref. [37].

The single crystal structure determination of [XeF_5_]_4_[Sn_5_F_24_] reveals that it is built of two-dimensional ([Sn_5_F_24_]^4−^)_∞_ grids and the [XeF_5_]^+^ cations located between them. The two-dimensional grids have a wave-like conformation (Figure 9). The ([Sn_5_F_24_]^4−^)_∞_ layer contains, both six- and seven-coordinated Sn(IV) interconnected by bridging fluorine atoms (Figure 9). It is a unique example of Sn(IV)-fluoride compound that does not consist just of [SnF_6_]^2−^ anions. The coordination of Sn(IV) by seven fluorine atoms is unprecedented.

**Figure 10:**
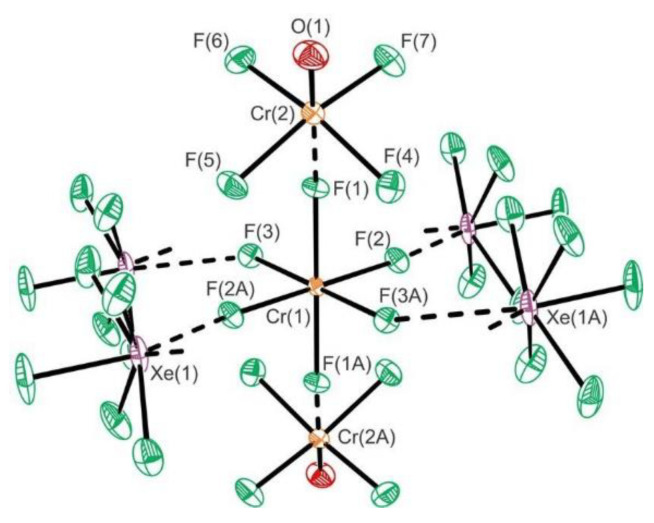
The coordination sphere of the [CrF_6_]^2−^ in the crystal structure of [XeF_5_]_2_[Cr^IV^F_6_]·2Cr^VI^OF_4_. Secondary Xe···-F and Cr···F bonding interactions are indicated by dashed lines. Copyright (2019) Wiley. Used with permission from Ref. [38].

Reactions between molten mixtures of XeF_6_ and Cr^VI^OF_4_ proceeds by F_2_ elimination to form [XeF_5_][Xe_2_F_11_][Cr^V^OF_5_]·2Cr^VI^OF_4_, [XeF_5_]_2_[Cr^IV^F_6_]·2Cr^VI^OF_4_, [Xe_2_F_11_]_2_[Cr^IV^F_6_], and [XeF_5_]_2_[Cr^V^_2_O_2_F_8_] [38]. On the other side, their reactions in aHF and CFCl_3_/aHF yield [XeF_5_]_2_[Cr^V^_2_O_2_F_8_]·2HF and [XeF_5_]_2_[Cr^V^_2_O_2_F_8_]·2XeOF_4_ [38]. Their crystal structures contain [XeF_5_]^+^ and/or [Xe_2_F_11_]^+^ cations, which interact with their respective anions by secondary Xe···F interactions. In the case of [XeF_5_][Xe_2_F_11_][Cr^V^OF_5_]·2Cr^VI^OF_4_ and [XeF_5_]_2_[Cr^IV^F_6_]·2Cr^VI^OF_4_, the CrOF_4_ is introduced into the coordination sphere of [Cr^V^OF_5_]^2−^ and [Cr^IV^F_6_]^2−^ anions, respectively, upon crystallizations (Figure 10). The crystal structure of [Xe_2_F_11_]_2_[Cr^IV^F_6_] is comprised of [Xe_2_F_11_]^+^ cations and [Cr^IV^F_6_]^2−^ anions.

**Figure 11:**
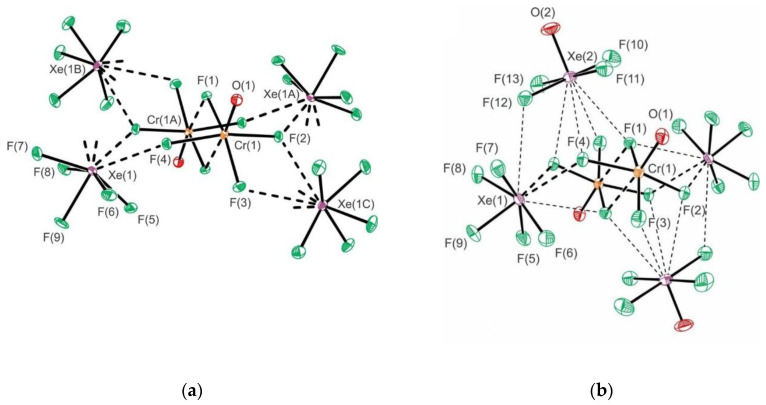
(**a**) The coordination sphere of the [Cr_2_O_2_F_8_]^2−^ anion in the crystal structure of [XeF_5_]_2_[Cr^V^_2_O_2_F_8_]. Secondary Xe···F bonding interactions and Cr···F_b_ bridge bonds are indicated by dashed lines; (**b**) The coordination environment around the [Cr^V^_2_O_2_F_8_]^2−^ anion in the crystal structure of [XeF_5_]_2_[Cr^V^_2_O_2_F_8_]·2XeOF_4_. Secondary Xe···F bonding interactions and Cr···F_b_ bridge bonds are indicated by dashed lines. Copyright (2019) Wiley. Used with permission from Ref. [38].

The crystal structures of [XeF_5_]_2_[Cr^V^_2_O_2_F_8_], [XeF_5_]_2_[Cr^V^_2_O_2_F_8_]·2HF, and [XeF_5_]_2_[Cr^V^_2_O_2_F_8_]·2XeOF_4_ contain [Cr^V^_2_O_2_F_8_]^2−^ anions, where the latter represents a new structural motif among the known oxyfluoroanions of Group 6 (Figure 11) [38].

**Figure 12:**
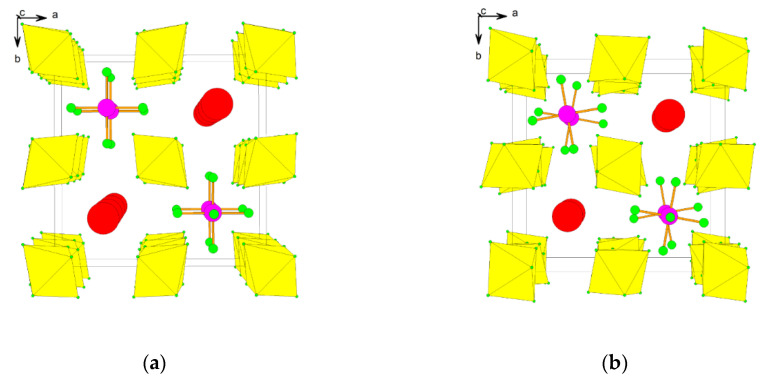
(**a**) Packing of Rb^+^ and [XeF_5_]^+^ cations and [SbF_6_]^−^ anions in the crystal structure of high-temperature (296 K) form of β-Rb[XeF_5_][SbF_6_]_2_; (**b**) Packing of Rb^+^ and [XeF_5_]^+^ cations and [SbF_6_]^−^ anions in the crystal structure of low-temperature (150 K) modification of α-Rb[XeF_5_][SbF_6_]_2_. Copyright (2017) Wiley. Used with permission from Ref. [32].

Reactions between ASbF_6_ (A = Rb, Cs) and [XeF_5_][SbF_6_] in aHF in 1:1 molar ratios yielded A[XeF_5_][SbF_6_]_2_ compounds upon crystallization [32]. A similar reaction between CsBiF_6_ and [XeF_5_][SbF_6_] (1:1) yielded mixed-cation/mixed-anion Cs[XeF_5_][Bi_x_Sb_1-x_F_6_]_2_ (x = ~0.37–0.39). The A[XeF_5_][SbF_6_]_2_ (A = Rb, Cs) and Cs[XeF_5_][Bi_x_Sb_1-x_F_6_]_2_ salts crystallize in two crystal modifications at low (α-phase, 150 K) and ambient (β-phase, 296 K) temperatures (Figure 12). High temperature modifications of A[XeF_5_][SbF_6_]_2_ (A = Rb, Cs) and Cs[XeF_5_][Bi_x_Sb_1-x_F_6_]_2_ are structurally related, while low-temperature modifications crystallize isotopically.

**Figure 13:**
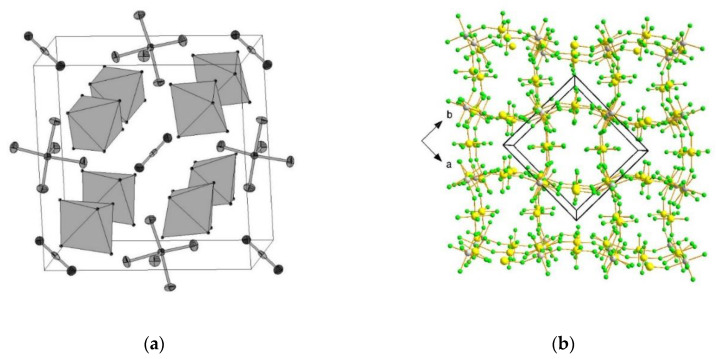
(**a**) Packing of [NO_2_]^+^ and [XeF_5_]^+^ cations, and [SbF_6_]^−^ anions in the crystal structure of NO_2_XeF_5_(SbF_6_)_2_; Only one of the two orientations of the disordered [SbF_6_]^−^ anions is depicted. Copyright (2015) Wiley. Used with permission from Ref. [39]; (**b**) The tri-dimensional [M(SbF_6_)_3_]^−^ framework with the monoclinic unit cell in the crystal structure of α-XeF_5_M(SbF_6_)_3_ (150 K; M = Mn) isotypic to M^2+^ = Mg, Co, Ni, Zn, and Cu compounds. The [XeF_5_]^+^ cations located within the cavities are omitted for clarity. Copyright (2016) Wiley. Used with permission from Ref. [40].

**Figure 14:**
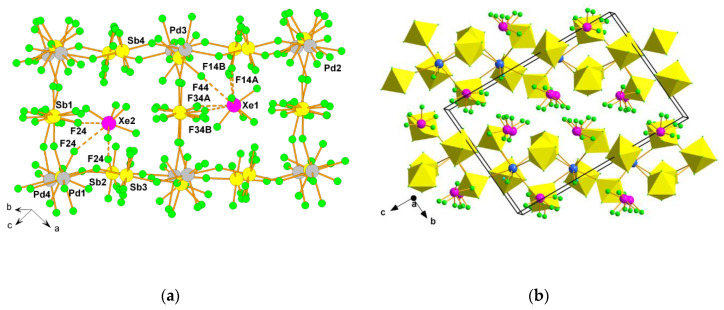
(**a**) Part of the crystal structure of β-XeF_5_Pd(SbF_6_)_3_ (296 K). Disorder of [XeF_5_]^+^ cations is not depicted. Only F atoms involved in secondary Xe···F contacts are labeled; (**b**) Packing of the columns in the crystal structure of (XeF_5_)_3_[Hg(HF)]_2_(SbF_6_)_7_ with a triclinic unit cell. Copyright (2016) Wiley. Used with permission from Ref. [40].

Reactions between XeF_5_SbF_6_ and NO_2_SbF_6_ or Cu(SbF_6_)_2_, respectively, at ambient temperature in aHF as a solvent yielded the first [XeF_5_]^+^/metal and [XeF_5_]^+^/non-metal mixed-cation salts, i.e., NO_2_XeF_5_(SbF_6_)_2_ and XeF_5_Cu(SbF_6_)_3_ (Figure 13) [39]. Further investigations led to six new examples of such type of compounds, i.e., XeF_5_M(SbF_6_)_3_ (M = Ni, Mg, Zn, Co, Mn and Pd) [40]. Additionally, single crystals of (XeF_5_)_3_[Hg(HF)]_2_(SbF_6_)_7_ were grown and crystal structure determined (Figure 14) [40]. For XeF_5_M(SbF_6_)_3_ salts, no phase transition was observed for compounds with M^2+^ (M = Ni, Mg, Cu, Zn, Co) that are smaller than Mn^2+^. The XeF_5_Mn(SbF_6_)_3_ and XeF_5_Pd(SbF_6_)_3_ crystallize in the low-temperature (Mn at 150 K and Pd at 260 K; α-phase) and high-temperature modifications (296 K; β-phase). The crystal structures of XeF_5_M(SbF_6_)_3_ (M = Ni, Mg, Cu, Zn, Co) and α- XeF_5_Mn(SbF_6_)_3_ are isotypic. The main feature of their crystal structures is the tri-dimensional framework consisting of the M^2+^ cations interconnected by the SbF_6_ octahedra, forming cavities within which the [XeF_5_]^+^ cations are located (Figure 13).

Although crystal structures of β-XeF_5_Mn(SbF_6_)_3_ and α- and β-modifications of XeF_5_Pd(SbF_6_)_3_, with larger M^2+^ cations, differ from the other XeF_5_M(SbF_6_)_3_ compounds the main structure motif is preserved, i.e., a three-dimensional (3D) framework constructed of the M^2+^ cations and SbF_6_ units and [XeF_5_]^+^ cations located inside the cavities (Figure 14).

### 2.3. Xenon(VI) Oxide Compounds

One of the main obstacles in the research of XeO_3_ is its extreme sensitivity. Solid XeO_3_ detonates when subjected to mild thermal or mechanical shock. For these reasons, experiments are usually limited to small quantities of XeO_3_ and its compounds (up to 20 mg).

**Figure 15:**
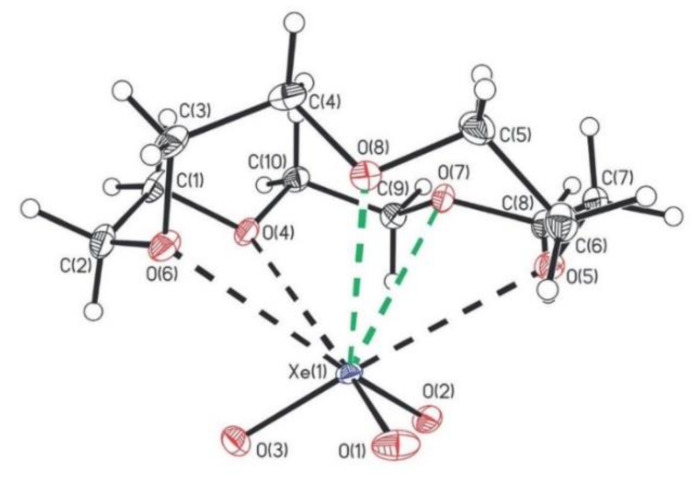
Side-on view of the structural unit in the crystal structure of (CH_2_CH_2_O)_5_XeO_3_. Copyright (2018) Wiley. Used with permission from Ref. [41].

The 15-crown adduct of XeO_3_, i.e., (CH_2_CH_2_O)_5_XeO_3_ was synthesized at ambient temperature by reaction of 15-crown-5 with HF-acidified aqueous solution of XeO_3_ [41]. It was structurally characterized by Raman spectroscopy and single-crystal X-ray diffraction (Figure 15).

**Figure 16:**
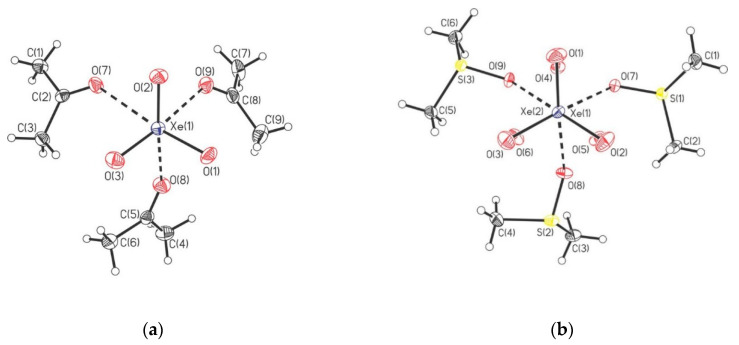

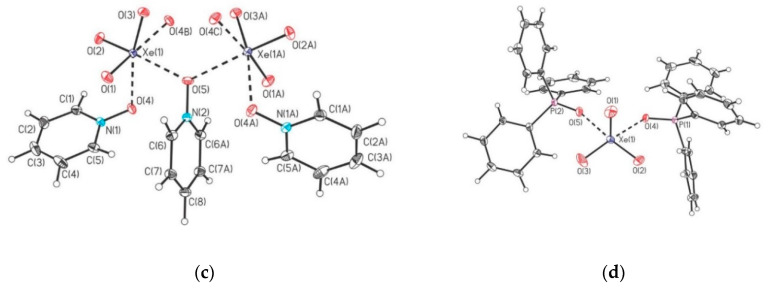
The structural units in the crystal structures of (**a**) [(CH_3_)_2_CO]_3_XeO_3_; (**b**) [(CH_3_)_2_SO]_3_(XeO_3_)_2_; (**c**) (C_5_H_5_NO)_3_(XeO_3_)_2_; (**d**) [(C_6_H_5_)_3_PO]_2_XeO_3_. Copyright (2019) Wiley. Used with permission from Ref. [42].

The crystalline adducts of XeO_3_ with triphenylphosphine oxide, dimethylsulfoxide (DMSO), pyridine-N-oxide, and acetonetriphenyl were prepared and characterized by low-temperature, single-crystal X-ray diffraction and Raman spectroscopy [42]. In [(CH_3_)_2_CO]_3_XeO_3_ each of the XeO_3_ molecules is coordinated to three acetone molecules (Figure 16). The structural unit of [(CH_3_)_2_SO]_3_(XeO_3_)_2_ is comprised of three DMSO ligands that bridge two XeO_3_ molecules (Figure 16), whereas in (C_5_H_5_NO)_3_(XeO_3_)_2_ two molecules of XeO_3_ are O-bridged by C_5_H_5_NO ligand and are each O-coordinated to a terminal C_5_H_5_NO (Figure 16). The [(C_6_H_5_)_3_PO]_2_XeO_3_ (low- and high-temperature modification) provides the only example of a five-coordinate XeO_3_ adduct which has only two secondary Xe···O bonding interactions (Figure 16).

**Figure 17:**
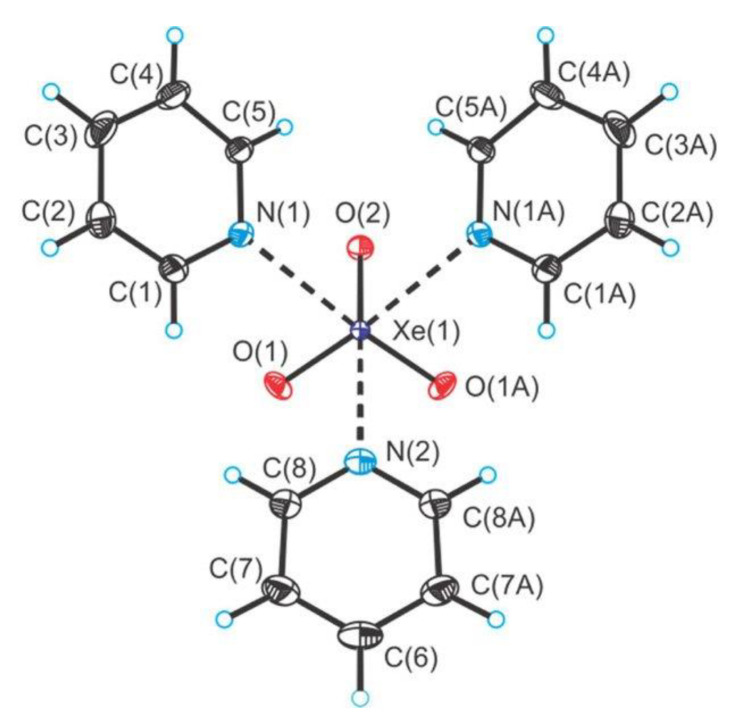
Top-down view of (C_5_H_5_N)_3_XeO_3_ in the X-ray crystal structure of (C_5_H_5_N)_3_XeO_3_. Copyright (2018) Elsevier. Used with permission from Ref. [43].

**Figure 18:**
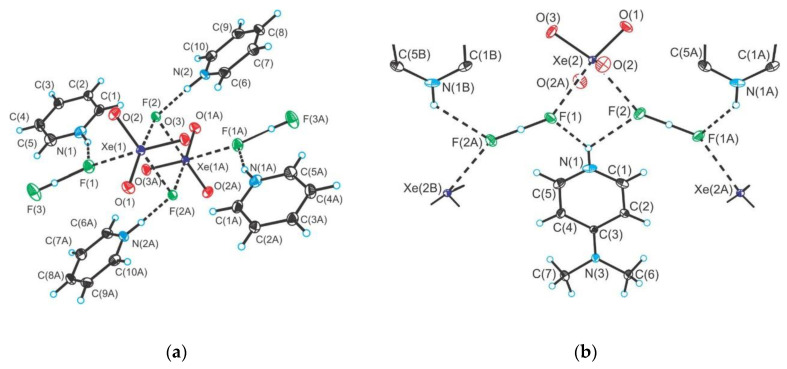
(**a**) The X-ray crystal structure of [C_5_H_5_NH]_4_[HF_2_]_2_[F]_2_(XeO_3_)_2_; (**b**) The X-ray crystal structure of [4-(CH_3_)_2_NC_5_H_4_NH][HF_2_]XeO_3_. Copyright (2018) Elsevier. Used with permission from Ref. [43].

Adducts between XeO_3_ and N-bases (pyridine and 4-dimethylaminopyridine) were also reported (Figure 17) [43]. Additionally, the crystal structures of the XeO_3_ adducts with the fluoride and bifluoride salts of their pyridinium cations were determined (Figure 18) [43].

## 3. Krypton

**Figure 19:**
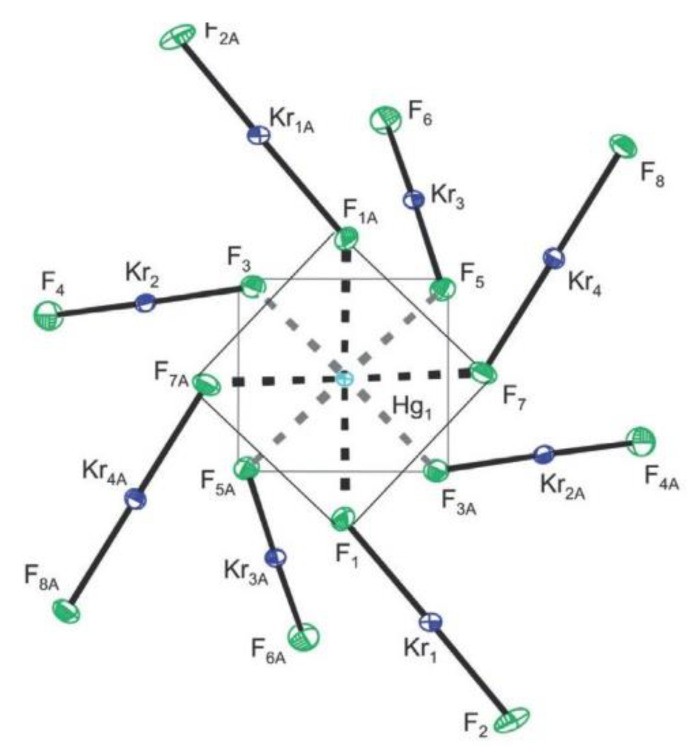
The [Hg(KrF_2_)_8_]^2+^ cation in the single-crystal X-ray structure of [Hg(KrF_2_)_8_][AsF_6_]_2_·2HF viewed down the *C*_2_ axis. Copyright (2018) Wiley. Used with permission from Ref. [45].

Beside xenon, krypton is the only noble gas where isolable compounds in macroscopic amounts can be prepared [44]. Its chemistry is limited to the +2 oxidation state. Known krypton(II) compounds are less stable than corresponding xenon(II) salts. Compounds in which KrF_2_ serves as a ligand towards Lewis centers have been only recently prepared [45]. The latest example is the synthesis and crystal structure determination of [Hg(KrF_2_)_8_][AsF_6_]_2_·2HF [45]. It is the first homoleptic KrF_2_ coordination complex of a metal cation (Figure 19).

**Figure 20:**
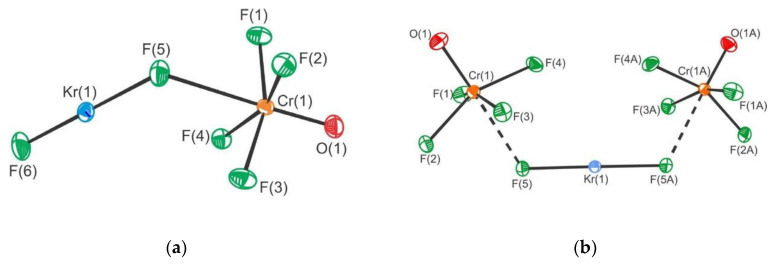
(**a**) The X-ray crystal structures of the near-staggered conformation of KrF_2_·CrOF_4_; (**b**) The structural unit in the X-ray crystal structure of syn-KrF_2_·2CrOF_4_. Copyright (2019) Wiley. Used with permission from Ref. [28].

The KrF_2_·CrOF_4_ and KrF_2_·2CrOF_4_ adducts were synthesized and their crystal structures determined [28]. The crystal structure of the former comprises of F–Kr–F···Cr(O)F_4_ species in which KrF_2_ is weakly coordinated to CrOF_4_ (Figure 20). In the latter both fluorine atoms of KrF_2_ coordinate to CrOF_4_ to form F_4_(O)Cr···F–Kr–F···Cr(O)F_4_.

## 4. Argon, Neon, Helium

In the gas phase all Ng elements including lighter representatives form an exceptionally large family of molecular species, ranging from fragile van der Waals adducts to strongly bound covalent species [2]. Some selected examples from the past include [Ar–N_2_]^+^ [46], HCCRg^2+^ (Rg = Ar and Kr) [47], ArCF_2_^2+^ [48] and NeH^+^ [49]. ArH^+^ was detected in the Crab Nebula, a supernova remnant. It was the first Ng molecule ever found in nature [50]. Charged Ng species, as for example (XeHXe)^+^, (KrHKr)^+^, and (KrHXe)^+^ [51], were synthesized in cold matrix.

### 4.1. Chemistry of Argon

Experimental chemistry of argon is limited to studies in the low-temperature matrices and molecules observed in the gas phase. The HArF is presently the only experimental known neutral molecule containing a chemically bound argon atom that is stable in a low temperature argon matrix [52]. Another story is the number of reported charged species detected in the gas phase. Some recent examples include observation of ArCH_2_^+^ in mass spectrometry experiments [53] and the production of ArOH^+^ molecular ion in a pulsed discharge/supersonic expansion of argon seeded with water vapor [54]. The cation complexes [ArB_3_O_4_]^+^, [ArB_3_O_5_]^+^, [ArB_4_O_6_]^+^, and [ArB_5_O_7_]^+^ were prepared via laser vaporization supersonic ion source in the gas phase [55]. Superelectophilic fragment anion [B_12_(CN)_11_]^−^, generated from the most stable gas-phase dianion [B_12_(CN)_12_]^2−^, binds argon covalently at room temperature [56].

### 4.2. Chemistry of Neon and Helium

Astrophysical detection of HeH^+^ in nearby interstellar space [57] is one of the greatest discoveries in molecular astrophysics. It was the first molecule to form after the big bang [58]. The synthesis of the isolable compounds containing neon and helium still remains an open challenge [8]. In 1992 it was reported that high pressure stabilizes the formation of a solid van der Waals compound of composition He(N_2_)_11_, obtained by compression of helium–nitrogen mixtures [59]. A few years ago, the discovery of non-inclusion compound Na_2_He at pressures higher than 113 GPa was reported [60]. It was described as an electride, i.e., a crystal made of positively charged ionic cores and with strongly localized valence electrons playing the role of anions. Peculiar Na_2_He calls for making our definitions of “compound” more precise [8]. Defect perovskites (He_2-x_□_x_)(CaZr)F_6_ can be prepared by inserting helium into CaZrF_6_ at high pressure [61]. Despite these, helium and also neon have not been forced to form genuine chemical compounds in neutral entities to this day [8]. One of the first steps towards a stable neon compound is claimed to be the experimental observation of the molecular anion [B_12_(CN)_11_Ne]^−^ [62].

## 5. Bonding Motifs in Noble-Gas Compounds

Despite the inertness of noble gases, Ng chemistry is rich in a variety of species with different bonding motifs [2,4,63]. The bonding motifs are a consequence of the polarization of the spherical electronic cloud of the Ng atoms by binding partners and they range from very weak ‘dispersion’ to stronger ‘induced-dipole’ interactions resulting in neutral and ionic ‘complexes’ of the noble gases, whose character ranges from fragile van der Waals adducts to definitely stable compounds [2].

Clusters of Ng atoms are held together by dispersion forces [2]. In various monocoordinated Ng species [X(Ng)*n* (*n* ≥ 1)], the character of the occurring interactions may span from weakly bound van der Waals adducts, held together by dispersion forces, to strongly bound covalent species [2].

Dicoordinated (‘inserted’) compounds have a general formula XNgY (X different or the same as Y), the Ng atom being involved in definitely recognizable interactions with both X and Y [2]. They can be neutral or ionic. The bonding of the noble-gas hydrides with the common formula HNgY compounds can be described as (HNg)^+^Y^−^ where (HNg)^+^ is mainly covalently bonded and the interaction of the (HNg)^+^ and Y^−^ is strongly ionic [63]. The most frequently drawn picture of the chemical bonding in XeF_2_ is the molecular orbital approach involving three-center, four-electron bonds 3-center 4-electron bonding (3c–4e) [28,64,65]. A single bond is thus spread over the F-Xe-F system.

XeF_2_ is a fluoride ion donor and it can form everything from weakly bond complexes to XeF^+^ salts with ionic formulation. In the weakly bond complexes [66], the XeF_2_ geometry stays intact. In XeF_2_ adducts as XeF_2_·CrOF_4_ [28] there is a slight elongation of Xe–F_b_ (F_b_ = bridging F atom interacting with Cr) bond, while the Xe–F_t_ (F_t_ = terminal F atom) bond is slightly shortened [2]. The elongation (weakening) of Xe–F_b_ and shortening (strengthening) of Xe–F_t_ bond is observed also in [M(XeF_2_)_x_]^n+^[A]^n−^ compounds where XeF_2_ is coordinated only by one of its fluorine atoms [27,67]. The distortion of XeF_2_ is visible in its Raman spectrum. Its vibrational band at 497 cm^−1^ is replaced by two vibrational bands. One is higher and one lower than 497 cm^−1^ [67]. Numerous products of reactions between XeF_2_ and various fluorides are formulated as [XeF]^+^ salts [28]. Although their formulations imply a simple ionic nature, there is no complete F^−^ transfer between XeF_2_ and fluoride ion acceptor [28]. The [XeF]^+^ cation and its anion interact through Xe–F···M bridges and the difference between Xe–F_b_ and Xe–F_t_ bond lengths is much more pronounced. Structural and vibrational evidences for the ionization pathway XeF_2_ → XeF^+^ + F^−^ were obtained studying the XeF_2_/XeF_5_AsF_6_ system [68] where at least five distinct well-characterized phases exist in the phase diagram of the XeF_2_/XeF_5_AsF_6_ mixture, and each of them exhibit a more or less pronounced dissociation of XeF_2_ into the ionic [XeF^+^]···[A–F^−^] form (A = Lewis acid) [63].

The bonding in XeF_6_ has caused considerable controversy that is not completely resolved [69,70,71]. The XeF_6_ is the strongest fluoride ion donor among XeF_2_, XeF_4_, and XeF_6_. It forms a large number of [XeF_5_]^+^ and [Xe_2_F_11_]^+^ salts with a variety of Lewis acid fluorides and oxyfluorides [4,5]. The [XeF_5_]^+^ cation geometry may be described in terms of pseudo-octahedral AX_5_E VSEPR arrangements of five bond pairs (X) and the lone electron pair (E) around Xe (A) which give rise to a square-pyramidal geometry of Xe and five F atoms [38,72]. Although these compounds are ionic in their nature, the [XeF_5_]^+^ and [Xe_2_F_11_]^+^ cations are extensively associated with their anions through Xe···F (F belongs to the anion) secondary bonding interactions [73].

In XeO_3_ adducts, the xenon-ligand bonds may be described as predominantly electrostatic, (weakly covalent) interactions between the highly electrophilic σ-holes of the xenon atom and the electronegative ligand atom [42].

## 6. Conclusions

This review shows that noble-gas chemistry is still interesting not just to chemists (at least to some of us) but also to astronomers, geologists, etc. For decades, astronomers have pursued for helium hydride HeH^+^, made of the two most common elements in the universe. In the laboratory the ion was discovered in 1925 [74]; however, we had to wait almost 100 years for the confirmation of the existence of HeH^+^ in nearby interstellar space [57]. High-pressure and high-temperature (by laser-heating) study of the possible formation of stable compounds between Ar and Ni at thermodynamic conditions representative of the Earth’s core resulted in the formation of ArNi compound (at 140 GPa and 1500 K) [75]. This result implies that the presence of argon in the Earth’s core is highly probable [75].

Recent review about the noble-gas/noble-metal chemistry suggests an inflation of such complexes, not only in theory or in microscopic amount in cryogenic situation, but also in large-scale syntheses [76].

Additional stimulation in the noble-gas research represents the discovery of noble gas (or aerogen) bonding [77]. It is a novel type of weak attractive noncovalent interaction [78]. According to IUPAC is defined as the attractive interaction between an electron rich atom or group of atoms and any element of Group-18 acting as electron acceptor [79,80].
